# p53 Regulation by TRP2 Is Not Pervasive in Melanoma

**DOI:** 10.1371/journal.pone.0087440

**Published:** 2014-01-27

**Authors:** Roland Houben, Corinna P. Schmid, Melissa Maier, Marion Wobser, Stephanie Motschenbacher, Jürgen C. Becker, Claudia S. Vetter-Kauczok, Gerhard Weyandt, Sonja Hesbacher, Sebastian Haferkamp

**Affiliations:** 1 Department of Dermatology, Venereology and Allergology, University Hospital Würzburg, Würzburg, Germany; 2 Department of General Dermatology, Medical University of Graz, Graz, Austria; University of Queensland Diamantina Institute, Australia

## Abstract

p53 is a central tumor suppressor protein and its inhibition is believed to be a prerequisite for cancer development. In approximately 50% of all malignancies this is achieved by inactivating mutations in the p53 gene. However, in several cancer entities, including melanoma, p53 mutations are rare. It has been recently proposed that tyrosinase related protein 2 (TRP2), a protein involved in melanin synthesis, may act as suppressor of the p53 pathway in melanoma. To scrutinize this notion we analyzed p53 and TRP2 expression by immunohistochemistry in 172 melanoma tissues and did not find any correlation. Furthermore, we applied three different TRP2 shRNAs to five melanoma cell lines and could not observe a target specific effect of the TRP2 knockdown on either p53 expression nor p53 reporter gene activity. Likewise, ectopic expression of TRP2 in a TRP2 negative melanoma cell line had no impact on p53 expression. In conclusion our data suggest that p53 repression critically controlled by TRP2 is not a general event in melanoma.

## Introduction

Advanced melanoma is a cancer that is largely resistant to cytotoxic drugs or irradiation; this had been at least in part attributed to an impaired p53 dependent apoptosis response [1]. The main biological functions of the transcription factor p53 include regulation of cell cycle progression, apoptosis, senescence, cellular differentiation and DNA repair [2]. Due to fast ubiquitination by the E3 ligase MDM2 and subsequent proteasomal degradation, p53 is frequently undetectable in normal cells [3]. However, upon several different stresses, including oncogenic stress or DNA damage, the amount of p53 is increased due to disruption of its degradation [4], [5]. p53 is a prototypic tumor suppressor and inactivating mutations in the p53 gene are present in approximately half of all human cancers [2]. Alternative mechanisms of p53 inactivation include MDM2/4 overexpression [3], [4], inhibition by viral oncoproteins [5] and loss of p14^ARF^, which binds MDM2 and inhibits its E3 ubiquitin ligase activity [3]. In addition, the transcriptional activity of p53 can also be impaired on a post-translational level by competition for DNA-binding sites by the p73 isoform ΔNp73 [6], [7], functional antagonism with the inhibitor of apoptosis-stimulating protein (iASPP) [6], [8] and sequestration in the cytosol by the ubiquitin ligase Parc [9]. Moreover, inhibition of p53 expression by tyrosine-related protein 2 (TRP2) has been proposed as a mechanism of p53 restriction [10]. Importantly, all of the aforementioned p53 inactivation mechanisms except inhibition by viral oncoproteins have been described in melanoma cells. This is of particular interest since p53 is frequently expressed in melanoma but inactivating mutations are rarely detectable [11]–[14]. Interestingly, the putative negative p53 regulator TRP2 is a marker of the melanocytic lineage and is expressed in more than 80% of metastatic melanoma lesions [15]. TRP2 is transcriptionally regulated by microphthalmia transcription factor (MITF) and Sox10. TRP2 acts in the melanin synthesis pathway downstream of tyrosinase catalysing the conversion of dopachrome to 5,5-dihydroxyindole-2-carboxylic acid (DHICA) [16]–[18]. Beside its function in melanin synthesis TRP2 has been proposed to regulate neural progenitor cell proliferation and to function as a pro-survival and anti-apoptotic molecule [19]. In melanoma cell lines an impaired apoptotic response after radiation or treatment with chemotherapeutic agents was found to be associated with increased expression of TRP2 [20]. A possible mechanistic explanation for the anti-apoptotic property of TRP2 has recently been described [10]. Sendoel and co-workers showed in *Caenorhabditis elegans* that secretion of the TRP2 homolog Tyr2 by sensory neurons inhibits p53 expression of germ cells. Moreover, TRP2 knockdown in WM266-4 melanoma cells lead to increased p53 expression sensitizing the cells to cisplatin-induced apoptosis.

In this report we further scrutinize a possible role of TRP2 in the regulation of p53 in five different melanoma cell lines and do not find such a relation.

## Materials and Methods

### Ethics Statement

Tumor samples from primary and metastatic melanomas were obtained by surgical excision for either therapeutic or diagnostic purposes and had undergone routine histology. The Institutional Review Board of Würzburg University Hospital approved all described studies and waived the need for written consent for histochemical analysis of anonymised tumor samples. Generation of the WueMel 45 melanoma cell line was done after written consent from the patient (Ethikkommission der Medizinischen Fakultät der Universität Würzburg; sequential study number 169/12).

### Tumor material and Tissue microarray (TMA)

After anonymization of tissue samples a dermatohistopathologist (C.K.) reviewed slides from all blocks, selecting representative areas of tumor tissue to be cored for generation of TMA as previously described [21]. The TMA used in this study contained 152 unique cases of primary melanoma (n = 129), and metastatic melanoma (n = 23). Additionally, 20 melanoma tissues (10 primary and 10 metastatic melanoma) for which a p53 wild type status had been confirmed by sequencing of the exon 5–8 were included in these studies.

### Immunohistochemistry (IHC)

4 µm sections of paraffin-embedded tumors and TMA were dried at 56°C and then treated twice with xylene for 10 min at room temperature. Subsequently, sections were washed twice with absolute ethanol and twice with 70% ethanol followed by one rinse with bi-distilled water. For antigen retrieval, sections were incubated with citrate buffer pH 9.0 (DAKO, Hamburg, Germany) for 10 min at 90°C and rinsed with bi-distilled water. Next, slides were rinsed twice with phosphate-buffered saline (PBS) and thereafter incubated with Blocking Solution (DAKO, S2023) for 10 min at room temperature. After two additional washing steps with PBS for 10 min at room temperature, the monoclonal α-p53 antibody (DO-7, DAKO) or α-TRP2 antibody (D18, Santa Cruz) was added to the sections at a predetermined concentration in PBS, followed by an over night incubation at 4°C. After two 10 min washes in PBS, biotinylated multispecies-specific secondary antibody (DAKO, K5003) was added to the sections for 30 min at room temperature. Slides were then washed twice in PBS/bovine serum albumin, and bound antibodies were visualized using streptavidin-HRP (DAKO K5003) and Vector Vip (Vector Laboratories, Burlingame USA) as peroxidase substrate according to the manufacturer's guidelines. Finally, the nuclei were stained with hemalaun.

### Scoring of Immunohistochemistry staining results

The cores of specimens on the tissue microarray (TMA) slides were scored using a semi-quantitative scoring system where staining intensity and the proportion of stained tumor cells is taken into account [7]–[18]. Every tumor was given a score according to the intensity of staining (no staining  = 0, weak staining  = 1, moderate staining  = 2, strong staining  = 3) and the extent of stained cells (up to 10% = 1, 11–50% = 2, 51–80% = 3, 81–100% = 4). The final histology score was determined by multiplying the intensity scores with the extent of positivity scores of stained cells, yielding scores of 0, 1, 2, 3, 4, 6, 8, 9 or 12. Scoring of the samples was performed by two independent individuals.

### Statistical analysis

The relationship between the p53 and TRP2 expression was statistically analyzed using the Prism Graph software 5.0 (San Diego, CA). The coefficient of determination (R^2^) and the p value were determined applying linear regression analysis comparing p53 and TRP2 histology scores.

### Cell culture

One melanoma cell line (WueMel45) was generated in our lab from a melanoma metastasis obtained from a patient for therapeutic purposes. This cell line as well as the melanoma cell lines FM88 [22], MelU [23], MelJuso [24] and M26 [25] were grown in RPMI 1640 supplemented with 10% fetal calf serum. For all five cell lines all coding exons of the p53 gene were sequenced demonstrating p53 wild type status [13].

### Cloning

The shRNA vectors TRP2_shRNA #1, #2 and #3 were obtained by cloning the shRNA sequences 5′-GTGATTCAAACAACTAACAGA**TCAAGAG**TCTGTTAGTTGTTTGAATCACA**TTTTTTT**-3′, 5′-AAGGTTGGCAATTTCATGCTGT**TCAAGAG**ACAGCATGAAATTGCCAACCTT**TTTTTTT**-3′ and 5′-CCACCAGTGATTCGGCAGA**TCAAGAG**TCTGCCGAATCACTGGTGG**TTTTTTT**-3′, respectively (the sense strand is given), into the lentiviral vector KH1 [26]. A Scr shRNA construct was used as a control [27]. A TRP2 cDNA was amplified by PCR and inserted into the retroviral expression vector pIH [28]. To make the TRP2 mRNA insensitive to the TRP2_shRNA #2 (TRP2in) six silent mutations (5′- ACAG**C**ATGAA**GC**TGCC**C**AC**G**-3′; exchanged nucleotides displayed in bold) were introduced in the shRNA target sequence using the quick change mutagenesis kit (Stratagene, Waldbrunn, Germany).

### Lentiviral and retroviral infection

Infectious viruses were raised by transfecting HEK293T cells [29]. The lentiviral shRNA vectors and the pGreenFire p53 reporter construct (SBI, Mountain View, Canada) were transfected in combination with the helper constructs p59, p60 and p61. The retroviral vector pIH-TRP2insens was transfected in combination with the helper constructs pHIT60 and pHIT456. Two days following transfection, virus supernatants were harvested and filtered through 0.45 µm pore size filters. For infection, virus-containing supernatants were supplemented with 4 µg/ml polybrene and then added to the target cells overnight. Then medium was changed and the cells were cultured for 3 more days prior to subjecting lysates of the cells to further analysis. Pure populations carrying the pGreenFire reporter were selected by culturing the cells in the presence of puromycin.

### p53 reporter gene assay

The p53 reporter construct pGreenFire lentiviral vector codes for a puromycin resistence and for green fluorescent protein (GFP) under the control of a p53 responsive element (4× CGACATGCCCGGGCATGT). Following puromycin selection flow cytometry to monitor GFP expression in pGreenFire transduced cells was performed on a FACSCanto (BD, Heidelberg, Germany). Mean GFP fluorescence intensities were normalized to the relative presence of the reporter constructs in the infected cells as determined by real time PCR [13].

### Western blot

Cells were lysed in Laemmli buffer and proteins were resolved by SDS-polyacrylamid gel electrophoresis and transferred to nitrocellulose membranes. Following blocking for 1 h with PBS containing 0.05% Tween 20 and 5% powdered skim milk, blots were incubated overnight with the primary antibody, washed three times with PBS with 0.05% Tween 20, and then incubated with a peroxidase coupled secondary antibody. Following extensive washing, the bands were detected using a chemoluminescence detection kit (Roche Diagnostics, Mannheim, Germany). Antibodies to p53 (D-01, Santa Cruz, Heidelberg, Germany) TRP2 (D18, Santa Cruz, Heidelberg, Germany) and β-tubulin (Sigma, Ottobrunn, Germany) were used.

## Results

### TRP2 and p53 co-expression in melanoma

Prompted by a report demonstrating an inhibitory effect of TRP2 on p53 in a melanoma cell line [10] we examined the expression of both proteins in a large panel of 172 paraffin embedded primary and metastatic melanoma samples by IHC. For this study we utilized a tumor tissue microarray of 129 primary and 23 metastatic melanomas. In addition a series of consecutive formalin-fixed, paraffin-embedded sections of human primary (n = 10) and metastatic melanoma (n = 10) were included where p53 mutational status was determined by nested PCR amplification and direct sequencing of exons 5–8 which harbor 95% of the known p53 mutations [13]. In line with previous reports no p53 mutations could be detected (data not shown) [13], [30].

**Figure 1:**
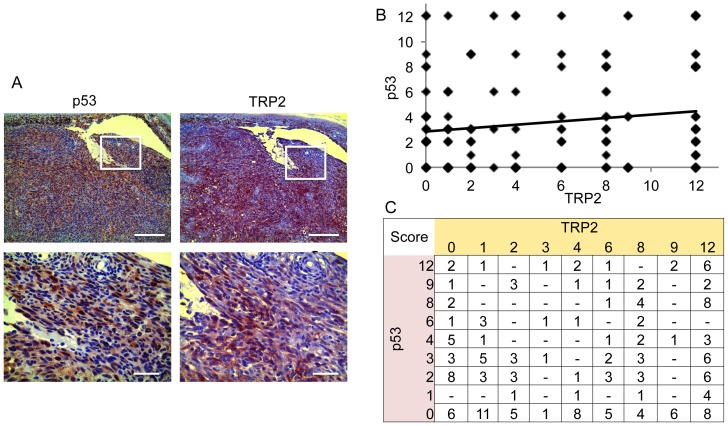
TRP2 and p53 expression in melanoma tissue. (A) Representative immunohistochemical staining for TRP2 and p53 in consecutive sections of a primary melanoma; scale bars: upper panel 1 mm, lower panel 100 µm. (B) Linear regression analysis of p53 and TRP2 histology scores derived from the analysis of 172 melanomas (139 primary and 33 metastatic melanoma) evaluated by a histopathologist. Staining intensity was scored between 0 and 3 and the extent of positivity between 0 and 4. By multiplying both values a minimum score of 0 and a maximum of 12 was derived; linear regression analysis comparing p53 and TRP2 histology scores revealed borderline-significant (p = 0,352) positive correlation and a coefficient of determination (R^2^) of 0,0258. (C) Since the number of individual dots in B cannot be visualized the distribution of individual p53 and TRP2 histology scores are displayed in the table.

Overall p53 expression was detectable (score ≥1) in 99 out of 139 (71,2%) cases of primary melanoma and in 17 out of 33 (51,5%) cases of metastatic melanoma (Figure 1). p53 immunoreaction showed consistent prominent nuclear and light cytoplasmic staining pattern in all samples. TRP2 was detected in 119 out of 139 (85%) cases of primary melanoma and 26 out of 33 (78%) cases of metastatic melanoma displaying nuclear and cytoplasmic staining. To compare p53 and TRP2 expression a semi-quantitative immunoreactive score was determined by multiplying the percentage of positive cells (P) with the average staining intensity (I) using the formula Q = P x I [31], [32] (Figure 1C). Statistical regression analysis did not reveal any negative correlation between TRP2 and p53 expression (Figure 1B). Quite the contrary, a very poorly fitting coefficient of determination (R^2^ = 0,0258) and only borderline-significant (p = 0,352) positive correlation was observed. Moreover, of all 52 samples with very weak or no TRP2 staining (histology score of 0 or 1) 17 (33%) also displayed very weak or negative p53 staining (histology score of 0 or 1). On the other hand of all 120 samples with middle to strong TRP2 staining (histology score of 2–12) 44 (37%) displayed very weak or negative p53 staining (histology score of 0 or 1). Taken together these analyses reveal, that p53 expression is independent of the TRP2 expression level. Moreover, both proteins are detectable in the majority of samples and therefore expression is not mutually exclusive.

### TRP2 knockdown does not impact on p53 expression

**Figure 2:**
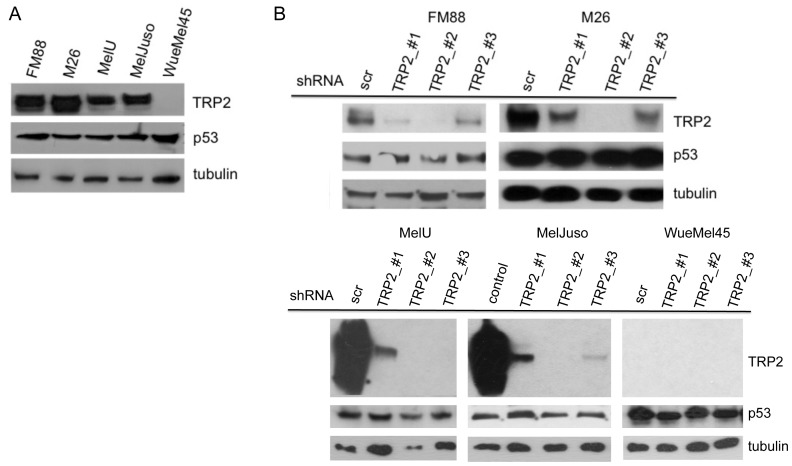
Knockdown of TRP2 by shRNA does not affect p53 expression level. (A) TRP2 and p53 expression in indicated melanoma cell lines was determined by western blot. Tubulin was used as a loading control. (B) The indicated melanoma cell lines were transduced with three different lentiviral TRP2 shRNAs (TRP2_#1, #2, #3) and on day four after shRNA infection the efficiency of the knockdown and p53 expression were analyzed by immunoblotting; a scrambled (scr) shRNA was used as a control.

To further scrutinize a possible role of TRP2 in the regulation of p53 we performed a series of TRP2 knockdown experiments addressing the expression and transcriptional activity of p53. Initially, we analyzed the expression of p53 and TRP2 in the melanoma cell lines FM88, M26, MelU, MelJuso, and WueMel45. p53 was present in all five melanoma lines, whereas TRP2 expression was detected by western blot in all cell lines except WueMel45. Three short hairpin RNAs were engineered to suppress TRP2 expression. The efficacy of TRP2 knockdown was confirmed by immunoblot; all three shRNAs strongly reduced TRP2 protein expression in melanoma cell lines and the knockdown was most prominent for TRP2-shRNA_#2 (Figure 2). To this end, even the most efficient TRP2 knockdown had no effect on p53 expression levels as determined by western blot (Figure 2).

**Figure 3:**
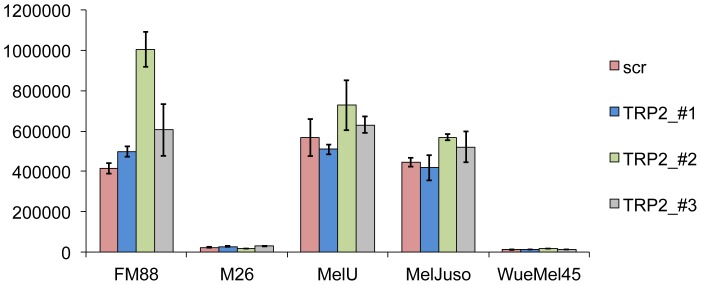
Effects of TRP2 inhibition by shRNA on transcriptional activity of p53. Indicated melanoma cell lines were stably transduced with a lentiviral pGreenFire reporter construct encoding for green fluorescence protein (GFP) under the control of a p53 responsive element (4× CGACATGCCCGGGCATGT). The cells were then infected with the different lentiviral supernatants carrying the shRNA expression construct KH1 containing either a scrambled or a sequence targeting TRP2. Mean GFP activity four days post infection is depicted, normalized to the relative vector load of the cell lines determined by Real time PCR.

To test whether the transcriptional activity of p53 may be altered by TRP2 knockdown, the set of melanoma lines were stably transduced with a reporter gene construct (pGreenFire) coding for GFP under the control of a p53 response element. The mean GFP expression was measured by flow cytometry and normalized for the relative number of incorporated pGreenFire copies as determined by real time PCR. As we have shown previously expression levels of p53 do not necessarily correlate with its transcriptional activity suggesting posttranslational inactivation of p53 [13] which has recently been shown to occur in melanoma e.g. by iASPP [8]. Accordingly, the M26 and WueMel melanoma cell lines show high p53 expression (Figure 2A) without any p53 reporter gene activity (Figure 3).

**Figure 4:**
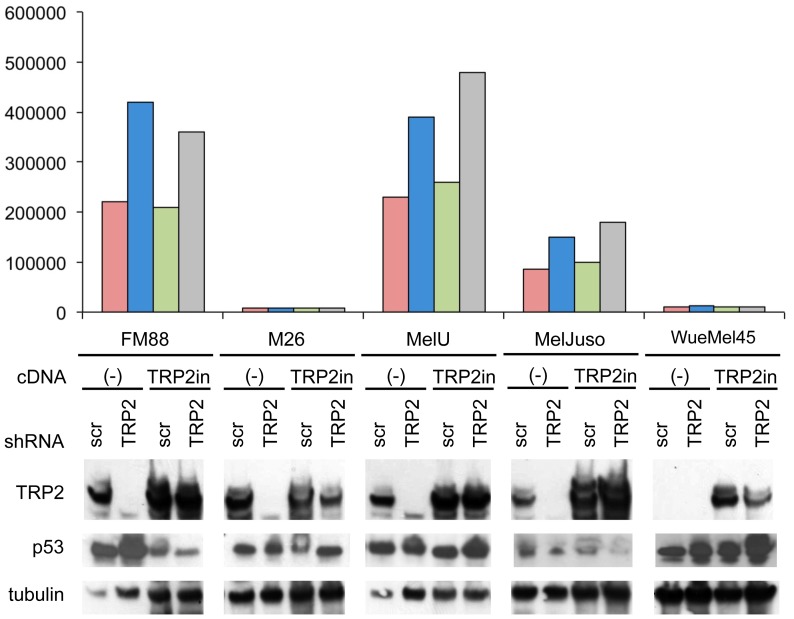
Ectopic re-expression of TRP2 does not rescue the p53 activation induced by TRP2-shRNA_#2. Indicated melanoma cell lines were stably transduced with a lentiviral p53 reporter construct and a modified TRP2 expression construct coding for TRP2 mRNAs in which the shRNA-binding site is modified by six silent mutations (TRP2in). On day 4 following infection total cell lysates were analyzed for TRP2 and p53 expression by immunoblotting (lower part) with tubulin used as a loading control. In the upper part the corresponding mean GFP fluorescence intensity is depicted, normalized to the relative reporter vector load of the cell lines determined by real time PCR.

Interestingly, TRP2 knockdown by two of the three shRNAs (#1 and #3) showed no impact on p53 reporter gene activity. However, infection with the TRP2-shRNA_#2 led to increased p53 reporter gene activity in FM88, Mel-U and MelJuso but not in M26 and WueMel 45 melanoma cells (Figure 3). Therefore, the results obtained with TRP2-shRNA_#1 and #3 suggest that TRP2 does not play a role in the regulation of p53 while induction of p53 reporter gene activity upon infection with the most effective TRP2-shRNA_#2 in some of the cells would be consistent with an inhibitory function of TRP2 in the p53 pathway. However, shRNAs notoriously exert off target effects [33]. To evaluate whether this is the case for TRP2-shRNA_#2 we investigated whether the observed p53 activation can be rescued by re-expression of TRP2. For this experiment we generated a modified TRP2 expression construct coding for an TRP2 mRNA in which the shRNA-binding site is modified by six silent mutations (TRP2in). When TRP2-shRNA_#2 was expressed no downregulation of TRP2 was observed in cells stably expressing the TRP2in construct thus, confirming the insensitivity of the ectopic TRP2in mRNA (Figure 4) to TRP2-shRNA_#2. Subsequent analysis of p53 transcriptional activity revealed that expression of this shRNA insensitive TRP2in could not revert the increase in p53 reporter gene activity in the presence of shRNA#2, indicating that this is only an off target effect. Moreover, ectopic expression of TRP2 in TRP2-negative WueMel45 melanoma cell line did not repress p53 expression or transcriptional activity as it would be expected when TRP2 functions as negative regulator in the p53 pathway (Figure 4).

## Discussion

Inactivation or at least partial repression of the p53 tumor suppressor pathway is thought to occur in almost all human cancers, and inactivating p53 mutations are the genetic alterations most frequently observed in malignancies [34], [35]. However, in several cancer entities, including melanoma p53 mutations are rare [36]. In these cancers with suppressed wild type p53 function, restoration of p53 activity is regarded as an attractive therapeutic strategy [35], [36]. However, for the development of such therapeutic approaches we need to understand the molecular mechanisms restricting p53 function in p53 wild type tumors. The great interest in such mechanisms in melanoma is reflected by several high impact publications during the last 12 months. Lu and co-workers, for example, demonstrated that in melanoma a cyclin B1/cdk1 protein complex phosphorylates iASPP leading to the inhibition of iASPP dimerization, increased nuclear entry of iASPP monomers, as well as binding and inhibition of p53 [8]. Gembarska and colleagues demonstrated that increased protein levels of the p53 inhibitor MDMX are frequently found in melanomas rendering the p53 tumor suppressor inactive [4]. A similar mechanism has previously been described for MDM2 [4], [37], [38]. Moreover, p53 function can be suppressed in melanoma due to expression of other p53 family members, including p63 and the N-terminal truncated isoforms ΔNp73 which both act dominant-negative via heterodimerization [6], [7], [39]. Most recently, a synonymous mutation in the BCL2L12 gene was identified in melanoma leading to increased expression of BCL2L12 which binds to p53 and inhibits its transcriptional activity [40].

In our study we investigated the role of the TRP2 in p53 regulation prompted by a report of Sendoel and colleagues who identified in *Caenorhabditis elegans* the TRP2 homolog TYR2 as a protein secreted by neurons and acting paracrine in neighboring germ cells to suppress CEP-1 (p53 homolog in the worm) dependent apoptosis [10]. Using the melanoma cell line WM266-4 which has been established from a lymph node metastasis [41], [42] they demonstrated that such a mechanism is evolutionarily conserved. In this respect they demonstrate that a TRP2 shRNA leads to upregulation of p53 protein levels and that the shRNA mediated TRP2 knockdown increased cisplatin induced apoptosis in the melanoma cell line [10]. To scrutinize to which extent TRP2 is restricting p53 expression and/or p53 function in melanoma we analyzed a large set of melanoma tissues as well as five melanoma cell lines. However, immunohistochemical analysis of the 172 melanoma lesions did not reveal any correlation between TRP2 and p53 expression, arguing against a critical dependency. Furthermore, neither efficient TRP2 knockdown in TRP2-positive melanoma cells using three different shRNAs caused a target specific increase in p53 function nor did overexpression of TRP2 in a TRP2 negative melanoma cell line repress p53 expression. The discrepancy between our data and the one reported by Sendoel et al. could be due to the fact that different cell lines were used in the two studies. Therefore, we cannot exclude that the inhibitory effect of TRP2 on p53 described by Sendoel et al. is a cell line specific phenomenon. It is also known that different cell culture practices and cell culture conditions used in different laboratories can have a major influence on gene expression and consequently can lead to different experimental results [43], [44]. Our data from cell culture experiments, however, are not only consistent between different cell lines and different shRNAs, but the conclusion derived from these experiments is also in line with our immunohistochemical studies. In summary our results suggest that TRP2 is not generally suppressing p53 in melanoma and that with respect to future therapeutic strategies the aforementioned proteins i.e. iASPP, p63 and MDMX appear to be more promising targets.
